# Intensive insulin therapy and mortality in critically ill patients

**DOI:** 10.1186/cc6807

**Published:** 2008-02-29

**Authors:** Miriam M Treggiari, Veena Karir, N David Yanez, Noel S Weiss, Stephen Daniel, Steven A Deem

**Affiliations:** 1 Department of Anaesthesiology, Box 359724, Harborview Medical Center, University of Washington School of Medicine, 325 Ninth Avenue, Seattle, WA 98104, USA; 2 Department of Pharmacy, Harborview Medical Center, 325 Ninth Avenue, Seattle, WA 98104, USA; 3 Department of Biostatistics, School of Public Health and Community Medicine, University of Washington, 1959 NE Pacific Street, Seattle, WA, 98195, USA; 4 Department of Epidemiology, School of Public Health and Community Medicine, University of Washington, Seattle, 1959 NE Pacific Street, Seattle, WA, 98195, USA

## Abstract

**Introduction:**

Intensive insulin therapy (IIT) with tight glycemic control may reduce mortality and morbidity in critically ill patients and has been widely adopted in practice throughout the world. However, there is only one randomized controlled trial showing unequivocal benefit to this approach and that study population was dominated by post-cardiac surgery patients. We aimed to determine the association between IIT and mortality in a mixed population of critically ill patients.

**Methods:**

We conducted a cohort study comparing three consecutive time periods before and after IIT protocol implementation in a Level 1 trauma center: period I (no protocol); period II, target glucose 80 to 130 mg/dL; and period III, target glucose 80 to 110 mg/dL. Subjects were 10,456 patients admitted to intensive care units (ICUs) between 1 March 2001 and 28 February 2005. The main study endpoints were ICU and hospital mortality, Sequential Organ Failure Assessment score, and occurrence of hypoglycemia. Multivariable regression analysis was used to evaluate mortality and organ dysfunction during periods II and III relative to period I.

**Results:**

Insulin administration increased over time (9% period I, 25% period II, and 42% period III). Nonetheless, patients in period III had a tendency toward higher adjusted hospital mortality (odds ratio [OR] 1.15, 95% confidence interval [CI] 0.98, 1.35) than patients in period I. Excess hospital mortality in period III was present primarily in patients with an ICU length of stay of 3 days or less (OR 1.47, 95% CI 1.11, 1.93 There was an approximately fourfold increase in the incidence of hypoglycemia from periods I to III.

**Conclusion:**

A policy of IIT in a group of ICUs from a single institution was not associated with a decrease in hospital mortality. These results, combined with the findings from several recent randomized trials, suggest that further study is needed prior to widespread implementation of IIT in critically ill patients.

## Introduction

Stress-induced hyperglycemia occurs frequently in critically ill patients and has been associated with increased morbidity and mortality in both diabetic and non-diabetic patients and in patients with traumatic injury [1-3], stroke [4-7], anoxic brain injury [8], acute myocardial infarction [9], post-cardiac surgery [10], and other causes of critical illness [11-13]. If causal, the mechanisms by which hyperglycemia affects outcomes could be related to suppressive effects on immune function and an associated increased risk of infection [14-16], endothelial damage [17], hepatocyte mitochondrial damage [18], and potentiation of tissue ischemia due to acidosis or inflammation [19,20].

Two observational [21,22] and two randomized [23,24] trials of surgical and medical critically ill patients have observed a higher incidence of favorable outcomes in critically ill patients treated with intensive insulin therapy (IIT) to achieve a blood glucose level of 80 to 110 mg/dL. However, other recently published studies suggest that there may be no benefit or even harm conferred by this approach in patients during cardiac surgery or recovering from cardiac arrest [25,26]. In addition, two recent randomized trials of IIT in critically ill patients were stopped early due to lack of benefit and hypoglycemia associated with IIT [27].

Although there is still debate whether the evidence is adequate to support a clear recommendation, the Institute for Healthcare Improvement [28] is recommending a care 'bundle' for severe sepsis which includes intensive glycemic control. Likewise, the Volunteer Hospital Association [29] uses glucose control as a quality indicator. As a result of these recommendations (which were made prior to the availability of the results from the most recent studies), tight glycemic control has increasingly become the standard of care for critically ill patients at our institution.

The objective of the present study was to investigate the effect of implementing a policy of tight glycemic control in a broader population of critically ill patients than previously studied, including a mix of trauma, surgical, neurosurgical, and medical intensive care unit (ICU) patients. To this end, we examined the outcomes of all patients admitted to the ICUs at Harborview Medical Center, a Level I trauma center and county hospital in Seattle, WA, before and after the introduction of intensive insulin protocols.

## Materials and methods

### Source population

Harborview Medical Center is a 374-bed municipal medical center affiliated with the University of Washington, Seattle, WA, and the only Level 1 trauma center in a five-state area (Washington, Wyoming, Alaska, Montana, and Idaho). There are seven ICUs located at Harborview Medical Center, serving a variety of patients with medical and surgical illness. The majority of patients are covered by ICU services with intensive staffing models, and all critical care protocols are implemented throughout all ICUs simultaneously. Nursing staffing ratios remained constant throughout the study period. For the purpose of this study, a cohort of all patients admitted to these ICUs over the course of a 4-year period between 1 March 2001 and 28 February 2005 was selected. All data were available from the hospital database originating from computerized medical and billing records and from a prospectively collected registry of trauma-related admissions [30]. The study was approved by the University of Washington Institutional Review Board, which waived the need for informed consent.

### Study design

The cohort was divided into three periods corresponding to changing glycemic control goals and insulin therapy protocols: period I, 1 March 2001 to 28 February 2002; during this period, there was no specific glycemic control protocol, and hyperglycemia was treated by a mix of subcutaneous and intravenous insulin, with a general target blood glucose of 120 to 180 mg/dL; period II, 1 March 2002 to 30 June 2003 (target blood glucose of 80 to 130 mg/dL); and period III, 1 July 2003 to 28 February 2005 (80 to 110 mg/dL). Study period was considered as a surrogate of IIT and was used as the main predictor of interest. The intensive insulin protocols consisted of explicit target glucose ranges and of dosing orders for intravenous insulin by continuous infusion combined with intravenous boluses if necessary (Appendix 1). In addition, educational efforts were directed at physicians and nursing staff to emphasize the potential benefits of tight glycemic control and to alert practitioners to the protocols. There were no major changes in glycemic management on the acute care wards during the study periods. Three other critical care protocols designed to improve clinical outcomes were implemented at the study hospital in 2001–2003: (a) a procedure for invasive diagnosis of ventilator-associated pneumonia (fall of 2001), (b) a lung protective ventilation protocol (spring of 2002), and (c) a protocol for liberation from mechanical ventilation (spring of 2003). There were no changes in the indications for admission to the ICU during the study period.

For each patient, only the first ICU stay per hospitalization and the first hospital stay were included in this analysis. Patients were excluded if their ICU stay was shorter than 24 hours or if the ICU stay was not completed before the end of the study period during which they were admitted. Patients younger than 16 years of age were also excluded.

Blood glucose levels obtained closest in time to 6 a.m. were collected from the central laboratory analyzer. This convention was chosen based on reporting of glycemic control in the two large randomized trials of IIT in critically ill patients and to provide consistency in reporting given the potential inaccuracy of capillary point-of-care glucose measurements in critically ill patients [23,24,31]. For frequency of hypoglycemia, data were included from both central laboratory measurements and point-of-care glucose testing in order to capture all hypoglycemic events.

Patients were classified by the admitting ICU service (surgical, medical, coronary, neurosurgical, or burn) and by the type of admission (surgical versus medical). The latter was determined by which service the majority of time in the ICU was spent after admission. Surgical admissions include those patients who spent the majority of their ICU time on the surgical, neurosurgical, or burn ICU services. In addition, the proportion of patients admitted after trauma was determined.

### Statistical analysis

Primary outcome measures defined *a priori *included ICU and hospital mortality. Secondary safety outcome measures included occurrence of moderate (glucose of less than 65 mg/dL) and severe (glucose of less than 40 mg/dL) hypoglycemia at any time during the ICU stay. Additional *a priori*-specified secondary outcomes included evidence of organ dysfunction as measured by Sequential Organ Failure Assessment (SOFA) scores.

Confounders were selected on the basis of *a priori *knowledge to account for severity of disease and other risk factors. Severity of illness was measured by the Simplified Acute Physiology Score (SAPS) II and the Acute Physiologic Score (APS) of the APACHE (Acute Physiology and Chronic Health Evaluation) III score in the first 24 hours of ICU admission in all patients; these scores were calculated from information available in the electronic medical record. The Injury Severity Score was obtained in all patients admitted after traumatic injury from the Harborview Trauma Registry. Other potential confounders that were adjusted for included age, race, gender, comorbidities as defined by SAPS II, mechanical ventilation at ICU admission, and history of diabetes.

We evaluated the distribution of baseline characteristics and their association with the mortality among patients admitted to the ICU during each respective insulin protocol implementation period (study period), using one-way analysis of variance or frequency tables, as appropriate. As relative risks closely approximated odds ratios (ORs), results are presented as ORs. All *P *values are two-sided. Logistic regression was used to model mortality probabilities in patients admitted in each study period and to adjust for *a priori*-selected potential confounding factors. Simple logistic regression models that included the main predictor of interest (study period as a surrogate of IIT) were fitted initially. Period I was used as a reference prior to the implementation of insulin protocols. Adjusted models included the main predictor of interest, the *a priori*-specified confounders, significant predictors of mortality, and additional confounders.

To explore the suggestion that different populations may derive a variable degree of benefit from insulin therapy, sensitivity analyses were conducted with varying assumptions regarding the underlying study population, including subgroup analyses for patients who were in the ICU for 3 days or less or more than 3 days, and further restricting the analyses to surgical or medical populations and to patients who were admitted after trauma.

Organ dysfunction scores, as measured by SOFA scores, arise from a mixture of discrete and continuous processes, making them ill-suited for standard statistical methods of analysis (for example, linear regression). We analyzed the SOFA scores using a generalization of the limited dependent variable model developed by Tobin [32] called the tobit model. The tobit model simultaneously combines a probit regression for the discrete component of the outcome (for example, zero SOFA scores) and a normal error regression model for the continuous component of the outcome (for example, SOFA scores greater than zero). For a detailed exposition of the tobit model and some of its extension, see Amemiya [33]. As SOFA scores are highly skewed, we log-transformed SOFA scores greater than zero to make the data more normal. We computed robust standard error estimates of the tobit model's regression coefficients using the non-parametric bootstrap of Efron [34]. Hypothesis tests to investigate associations between predictor variables and SOFA scores were performed using Wald statistics. STATA statistical software (version 9.2; StataCorp LP, College Station, TX, USA) was used for all analyses. *P *values are two-sided.

## Results

### Study population and baseline characteristics

The study population consisted of 10,456 patients, of whom 2,366 were admitted during period I, 3,322 during period II, and 4,768 during period III. The number of trauma patients in the study cohort was 857 (36%) in period I, 1,203 (36%) in period II, and 1,920 (40%) in period III. The distributions of demographic and baseline characteristics of the patient population were broadly similar across the three periods (Table 1). Compared with patients admitted during period I, history of type I diabetes was less frequent in patients admitted in periods II and III, whereas type II diabetes was more common in the latter periods (Table 1). Blood glucose at ICU admission was lower in period III compared with the other two periods. The average SAPS II and APS III scores were lower in period III than in periods I and II. Requirement for mechanical ventilation at ICU admission was less frequent over time.

**Table 1:** Baseline characteristics at intensive care unit admission stratified by study period

Clinical characteristic	Period I (n = 2,366) 1 Mar 01 to 28 Feb 02	Period II (n = 3,322) 1 Mar 02 to 30 Jun 03	Period III (n = 4,786) 1 Jul 03 to 28 Feb 05
Age in years, mean ± SD	51.1 ± 18.5	51.6 ± 19.1	51.6 ± 19.0
Male gender, n (%)	1,467 (62.0)	2,071 (62.4)	3,044 (63.9)
Race or ethnic group, n (%)^a^			
Native American	70 (2.96)	62 (1.87)	105 (2.20)
Asian	142 (6.00)	220 (6.63)	294 (6.17)
African-American	224 (9.47)	291 (8.77)	369 (7.74)
Caucasian	1,651 (69.78)	2,310 (69.60)	3,422 (71.77)
Hispanic	91 (3.85)	156 (4.70)	228 (4.78)
Unknown	188 (7.95)	283 (8.52)	350 (7.34)
History of diabetes, n (%)			
Type I	64 (2.7)	68 (2.1)	77 (1.6)
Type II	211 (8.92)	367 (11.1)	574 (12.0)
History of chronic disease, n (%)^b^	74 (3.2)	95 (2.9)	137 (2.9)
SAPS II, mean ± SD^c^	39.1 ± 18.1	39.3 ± 18.9	37.2 ± 18.4
APS III, mean ± SD^d^	53.1 ± 21.8	53.1 ± 23.3	50.5 ± 23.3
Trauma patients, n (%)	857 (36)	1,203 (36)	1,920 (40)
Injury Severity Score, mean ± SD	21.2 ± 10.9	20.8 ± 11.1	20.7 ± 10.6
Type of admission, n (%)			
Surgical admission	1,440 (60.9)	2,003 (60.3)	3,084 (64.7)
Medical admission	926 (39.1)	1,319 (39.7)	1,684 (35.3)
Admitting service, n (%)	n = 2,366	n = 3,314	n = 4,745
Surgical ICU	759 (32.1)	1,061 (32.0)	1,604 (33.8)
Medical ICU	756 (31.9)	1,025 (30.9)	1,303 (27.5)
Coronary ICU	170 (7.19)	270 (8.2)	350 (7.4)
Neurosurgical ICU	607 (25.7)	871 (26.3)	1,335 (28.1)
Burn ICU	74 (3.1)	87 (2.6)	153 (3.2)
Weight in kilograms, mean ± SD	83.0 ± 24.9	84.6 ± 26.0	84.5 ± 25.5
Admission glucose in mg/dL, mean ± SD	162.5 ± 88.9	161.3 ± 82.5	152.1 ± 69.1
Mechanical ventilation, n (%)	1,424 (60.2)	1,878 (56.5)	2,578 (54.1)

^a^Race or ethnic group was assigned on the basis of hospital record. ^b^Chronic disease defined according to Simplified Acute Physiology Score (SAPS) II criteria. ^c^SAPS II can range from 0 to 162, with higher scores indicating a higher risk of death. ^d^APS is the acute physiology score of the APACHE (Acute Physiology and Chronic Health Evaluation) III score; it can range from 0 to 251, with higher scores indicating a higher risk of death. ICU, intensive care unit; SD, standard deviation.

Except for gender, ethnic group, history of diabetes I or II, and weight, all variables in Table 1 were associated with hospital mortality. In particular, the OR of hospital mortality for admission glucose was 1.0009 (95% confidence interval [CI] 1.0002, 1.0016; *P *= 0.014) for a 1 mg/dL increase in admission blood glucose.

### Insulin use, glucose control, and hypoglycemia

The proportion of patients receiving insulin infusion increased dramatically over the study periods, from 9% in period I to 25% in period II and then further to 43% in period III (Table 2). Due to the lower threshold to initiate insulin treatment, and the broadened exposure to insulin among patients without insulin resistance, patients (on average) received lower doses of insulin to control blood glucose in period III compared with the previous two periods. The average 6 a.m. blood glucose concentrations as measured in the central laboratory decreased from 144 mg/dL in period I to 139 mg/dL in period II to 129 mg/dL in period III. Moderate (<65 mg/dL) and severe (<40 mg/dL) hypoglycemic events increased approximately threefold to fourfold from the first to the third study periods (Table 2). There was excess mortality associated with hypoglycemia across the three time periods (Table 3). We explored the association between mean glucose and mortality restricting the analysis to period I (baseline). The OR of hospital mortality for mean blood glucose in period 1 was 1.0078 (95% CI 1.0043, 1.0112; *P *<0.01).

**Table 2:** Insulin usage, glucose control, and safety of an intensive insulin protocol stratified by study period

	Period I (n = 2,366) 1 Mar 01 to 28 Feb 02	Period II (n = 3,322) 1 Mar 02 to 30 Jun 03	Period III (n = 4,786) 1 Jul 03 to 28 Feb 05	*P *value^a^
Patients receiving insulin infusion, n (%)	206 (8.71)	817 (24.59)	2,027 (42.51)	<0.01
Patients receiving subcutaneous insulin, n (%)	307 (14.21)	349 (13.93)	237 (8.65)^c^	<0.01
Patients receiving any insulin, n (%)	513 (21.68)	1,166 (35.10)^b^	2,264 (47.48)^c^	<0.01
Average daily dose of insulin, units/24 hours, mean ± SD^d^	72.4 (84.9)	55.7 (58.7)	47.5 (43.1)	<0.01
Central lab 6 a.m. blood glucose	n = 2,339	n = 3,278	n = 4,643	
Mean ± SD	144.0 ± 38.1	138.7 ± 34.4	129.3 ± 30.3	<0.01
Median (IQR)	138 (120–160)	134 (117–153)	125 (112–140)	
Average daily blood glucose, mean ± SD	146.6 ± 41.5	142.0 ± 37.4	132.6 ± 30.8	<0.01
Average daily blood glucose, median (IQR)	139 (121–161)	136 (120–156)	128 (115–143)	
Average point-of-care blood glucose	n = 700	n = 1,407	n = 2,610	
Mean ± SD	167.4 ± 46.7	148.0 ± 36.8	131.6 ± 31.2	<0.01
Hypoglycemia <40 mg/dL, n (%)^e^	24 (1.01)	53 (1.60)	103 (2.15)^c^	<0.01
Hypoglycemia <65 mg/dL, n (%)^e^	115 (4.86)	351 (10.57)	810 (16.99)	<0.01
High-concentration glucose replacement, n (%)^f^	86 (3.63)	244 (7.34)	462 (9.69)	<0.01
Hyperglycemia >200 mg/dL, n (%)	330 (14.1)	367 (11.1)	339 (7.26)	<0.01

^a^Analysis of variance or chi-square comparing the three study periods; period I is used as reference. ^b^*P *<0.05 compared with period I (Wald test). ^c^*P *<0.01 compared with period I (Wald test). ^d^Patients receiving insulin infusion only. ^e^Number of patients with at least one episode of hypoglycemia; blood glucose values include both point-of-care and central laboratory measurements. ^f^High-concentration glucose indicates administration of 50 mL of dextrose 50% (25 g). IQR, interquartile range; SD, standard deviation.

### Mortality

Overall, the crude hospital mortality rates were 14.1% in period I, 15.7% in period II, and 14.4% in period III (Table 3). These figures are within the range predicted by severity of illness scores (SAPS II and APS III). After adjusting for age at admission, history of diabetes, SAPS II with age points removed, admitting service, and mechanical ventilation at ICU admission, the adjusted relative odds of hospital mortality were not significantly different for patients who were admitted during period II (OR 1.11, 95% CI 0.93, 1.31) or period III (OR 1.15, 95% CI 0.98, 1.32) compared with patients who were admitted during period I (Table 4). In a model that included admission blood glucose levels, the ORs of hospital mortality were 1.11 (95% CI 0.93, 1.31) in period II and 1.16 (95% CI 0.99, 1.37) in period III.

**Table 3:** Crude estimates of mortality, length of stay, and organ dysfunction stratified by study period

	Period I (n = 2,366) 1 Mar 01 to 28 Feb 02	Period II (n = 3,322) 1 Mar 02 to 30 Jun 03	Period III (n = 4,786) 1 Jul 03 to 28 Feb 05	*P *value^a^
ICU mortality, n (%)	214 (9.04)	358 (10.78)	465 (9.75)	0.086
OR (95% CI) of ICU mortality	1.00	1.21 (1.01, 1.46)	1.09 (0.92, 1.29)	
Hospital mortality, n (%)	334 (14.12)	522 (15.71)	686 (14.39)	0.157
OR (95% CI) of hospital mortality	1.00	1.13 (0.97, 1.32)	1.02 (0.88, 1.17)	
Patients in ICU <3 days	n = 1,296	n = 1,829	n = 2,678	
ICU mortality in patients in ICU <3 days, n (%)	76 (5.86)	128 (7.0)	181 (6.76)	0.428
Hospital mortality in patients in ICU <3 days, n (%)	122/1,174 (9.4)	203/1,829 (11.1)	283/2,678 (10.6)	0.310
Hospital mortality in patients with hypoglycemia <40 mg/dL, n (%)	9/24 (38)	23/53 (43)	41/103 (40)	0.863
Average SOFA score, mean ± SD^b^	1.65 (2.00)	1.77 (2.09)	1.62 (1.98)	<0.01
Maximum SOFA score, mean ± SD^b^	2.82 (2.85)	2.96 (2.99)	2.74 (2.84)	0.004

^a^Analysis of variance or chi-square comparing the three study periods; period I is used as reference. ^b^Scores for the Sequential Organ Failure Assessment (SOFA) can range from 0 to 24, with higher scores indicating a higher risk of death. CI, confidence interval; ICU, intensive care unit; OR, odds ratio; SD, standard deviation.

**Table 4:** Multivariable regression analysis (maximum likelihood estimation): intensive care unit and hospital mortality^a^

	Period II (n = 3,322)^b^ 1 Mar 02 to 30 Jun 03		Period III (n = 4,786)^b^ 1 Jul 03 to 28 Feb 05	
	OR (95% CI)	*P *value	OR (95% CI)	*P *value

Entire ICU population				

	n = 3,310		n = 4,739	

ICU mortality	1.20 (0.98, 1.47)	0.071	1.26 (1.04, 1.53)	0.019
Hospital mortality	1.11 (0.93, 1.31)	0.248	1.15 (0.98, 1.35)	0.088

Entire ICU population, ICU LOS ≤ 3 days				

	n = 1,808		n = 2,619	

ICU mortality	1.21 (0.84, 1.74)	0.317	1.65 (1.16, 2.33)	0.005
Hospital mortality	1.17 (0.87, 1.57)	0.288	1.47 (1.11, 1.93)	0.007

Entire ICU population, ICU LOS >3 days				

	n = 1,484		n = 2,033	

ICU mortality	1.21 (0.95, 1.54)	0.125	1.14 (0.90, 1.44)	0.268
Hospital mortality	1.08 (0.87, 1.33)	0.501	1.01 (0.83, 1.24)	0.918

^a^All estimates are adjusted for admission age, history of diabetes, Simplified Acute Physiology Score (SAPS) II with age points removed, mechanical ventilation at ICU admission, and admitting service. ^b^Period I is used as reference category for all analyses (n = 2,366 for the entire ICU population, n = 1,282 for patients in ICU ≤3 days, and n = 1,067 for patients in ICU >3 days). CI, confidence interval; ICU, intensive care unit; LOS, length of stay; OR, odds ratio.

When the models were re-fitted to analyze mortality with categories of ICU length of stay (LOS), the ORs of hospital mortality were significantly higher in patients with an ICU LOS of 3 days or less (Table 4). In contrast, in patients with an ICU LOS of greater than 3 days, there was no association between study period and mortality (Table 4). Hospital mortality was not decreased during period III in either the medical or surgical population (whether trauma patients or others) (Table 5).

**Table 5:** Multivariable regression analysis: intensive care unit and hospital mortality in population subgroups^a^

	Period I (n = 2,366) 1 Mar 01 to 28 Feb 02	Period II (n = 3,322) 1 Mar 02 to 30 Jun 03	Period III (n = 4,786) 1 Jul 03 to 28 Feb 05
	OR (95% CI)	OR (95% CI)	OR (95% CI)

Surgical and trauma ICU population			

	n = 1,429	n = 1,991	n = 3,011

ICU mortality	1.00	1.27 (0.97, 1.67)	1.40 (1.08, 1.82)^b^
Hospital mortality	1.00	1.18 (0.94, 1.48)	1.18 (0.95, 1.47)

Trauma ICU population^c^			

	n = 854	n = 1,195	n = 1,866

ICU mortality	1.00	1.25 (0.85, 1.84)	1.76 (1.23, 2.53)^d^
Hospital mortality	1.00	1.12 (0.81, 1.54)	1.16 (0.85, 1.57)

Medical ICU population			

	n = 920	n = 1,301	n = 1,641

ICU mortality	1.00	1.12 (0.83, 1.52)	1.10 (0.82, 1.47)
Hospital mortality	1.00	1.02 (0.87, 1.41)	1.11 (0.87, 1.41)

^a^All estimates are adjusted for admission age, history of diabetes, Simplified Acute Physiology Score (SAPS) II with age points removed, and mechanical ventilation at ICU admission. ^b^*P *<0.05 multivariable regression (maximum likelihood estimation). ^c^In the trauma ICU population, the models also adjust for Injury Severity Score. ^d^*P *<0.01 multivariable regression (maximum likelihood estimation). CI, confidence interval; ICU, intensive care unit; OR, odds ratio.

Overall, the crude ICU mortality rates were 9.0% in period I, 10.8% in period II, and 9.8% in period III. After adjustment, the ORs of ICU mortality were significantly higher comparing period III (OR 1.26, 95% CI 1.04, 1.53) with period I both in the entire population (Table 4) and in the subgroups of surgical and trauma patients (Table 5), but not in the medical population.

### Organ Dysfunction Score

Overall, the unadjusted mean SOFA score tended to decrease over time across the study periods (Table 3). However, after adjustment for imbalances in baseline characteristics, including age, history of diabetes, SAPS II with age points removed, admission blood glucose, and mechanical ventilation at ICU admission, there was a 0.028 (95% CI -0.004, 0.06; *P *= 0.082) increase in the mean of the natural logarithm SOFA score comparing patients in periods II and I and there was a 0.043 (95% CI 0.013, 0.073; *P *= 0.005) increase in the mean of the log SOFA score comparing patients in periods III and I.

## Discussion

The present study observed that implementation of IIT, with the percentage of patients receiving insulin by infusion increasing from 9% in period I to 42% in period III, was associated with no hospital mortality benefit. Furthermore, an incremental mortality increase associated with IIT was observed in patients with an ICU stay of 3 days or less. These latter findings are consistent with those from a similar subgroup in a randomized controlled trial (RCT) of IIT in medical ICU patients [24].

Our study has several potential limitations. Despite our effort to control for confounding by assessing patient characteristics across the three study periods and adjusting for any baseline differences seen, the possibility of bias remains. However, arguing in support of a true incremental mortality associated with tighter glucose control was that the trend observed was opposite of what might have been expected if confounding had been present, given that several other clinical protocols designed to improve patient outcomes were implemented in the ICUs of the study hospital at around the same time as the IIT protocols. Admittedly, two of these protocols would not be expected to have any appreciable effect on mortality (invasive diagnosis of ventilator-associated pneumonia and ventilator weaning protocol) and the third (a protocol for lung protective ventilation) was widely practiced prior to formal protocol release. We did not observe any mortality differences between the first and second halves of each study period, arguing against an overall trend of increasing in mortality during the study (data not shown). These arguments suggest that our approach to evaluating the impact of the implementation of an IIT protocol on mortality is a valid one.

The major strength of the current study lies in the large cohort of patients included (>10,000) and the availability of extensive clinical data that allowed adjustment for severity of illness across time periods.

Four prior published studies have observed at least some reduction in mortality associated with IIT in critically ill patients. Van den Berghe and colleagues [23] reported the results of a large randomized trial of IIT in patients admitted to a surgical ICU, approximately 60% of whom had undergone cardiac surgery. IIT (blood glucose range of 80 to 110 mg/dL) was associated with a reduction in mortality from 8.0% to 4.6% compared with conventionally treated patients (blood glucose range of 180 to 200 mg/dL) in addition to reductions in multiple morbidities [23]. In a subsequent study, van den Berghe and colleagues [24] randomly assigned patients admitted to a medical ICU to IIT or conventional blood glucose management. There was no clear mortality benefit for IIT in the intention-to-treat population, whether for ICU (24.2% IIT group versus 26.8% conventional treatment group) or hospital (37.3% IIT versus 40% conventional treatment group) mortality. However, IIT was associated with reduced in-hospital mortality in those patients who remained in the ICU for more than 3 days. In two studies that reported outcomes before and after implementation of an intensive glucose management protocol, mortality after implementation of the protocol was improved compared with prior to protocol implementation [21,22]. However, neither study adjusted for severity of illness or other factors that may have changed over time.

There are several reasons that may explain why the results of our study differ from these previous investigations of IIT. The implementation of progressively more aggressive insulin therapy protocols at our institution resulted in a large change in practice: the use of IIT rose from 9.6% of patients in period I to 42% in period III. Although this resulted in a reduction in mean daily and mean morning glucose concentrations, with a difference of approximately 15 mg/dL between periods I and III, we were unable to consistently achieve blood glucose concentrations within the range of 80 to 110 mg/dL. It is possible that the benefits of IIT are not achieved unless glucose concentrations are lower than 110 mg/dL.

Failure to achieve the targeted glucose levels reflects the difficulty in application of clinical protocols to real-world practice, outside the rigid confines of RCTs, and has been recognized previously [35-37]. Despite an explicit protocol combined with continuing educational efforts to alert physicians and nurses to the potential benefits of tight glycemic control, glucose levels (on average) remained approximately 20 mg/dL above the target range. This 'failure' likely occurred at several levels, although we are unable to discern specific causes within the limitations of our study design.

A major difference between our cohort and that included in the trials of van den Berghe and colleagues [23,24] is the form of nutritional support used. In the studies of van den Berghe and colleagues, nutritional support was very aggressive: parenteral nutrition was administered early in the course of care and comprised the vast majority of non-protein calories during the first few days of ICU stay. This is in contrast to the practice at our institution, where parenteral nutrition generally is not instituted until 3 to 5 days after ICU admission. Parenteral nutrition reduces endogenous glucose production and promotes hyperglycemia in critical illness, effects which may be modulated by insulin administration [38]. It is not clear how these effects would translate into increased benefit from IIT.

Finally, our study differs from the trials of van den Berghe and colleagues [23,24] in that we examined a mixed population of critically ill patients, with approximately 60% of patients carrying a surgical diagnosis at admission, with a high representation of trauma patients (approximately 56% of the entire study cohort), including those with neurological injury. Our data suggest that, if anything, ITT was associated with an increased mortality among trauma patients, although the reasons for this are not clear. Previous retrospective studies have found an association between hyperglycemia and mortality in trauma patients [1,2,39-43] and possibly a lower mortality temporally associated with the implementation of an IIT protocol and reduction in glucose variability [22,44]. However, our study is the first to examine the effects of IIT on outcome in this population, applying rigorous adjustments for patients' baseline characteristics. Cardiac surgical services are not performed at out institution; therefore, our surgical population differs from that of the study of van den Berghe [23], in which the majority of patients were recovering from cardiovascular surgery.

Several other studies also did not observe evidence of benefit of ITT. A recently published multicenter RCT in 537 patients with severe sepsis found no difference in mortality or organ failure in IIT versus conventional glucose management groups [45]. The incidence of severe hypoglycemia was increased in patients randomly assigned to IIT (17.0% versus 4.1%). Another recent study investigated the effect of intraoperative IIT on the outcome of patients undergoing cardiac surgery [46]. Patients were randomly assigned to IIT (glucose range of 80 to 100 mg/dL) or conventional treatment (glucose of less than 200 mg/dL). The study reported higher mortality and higher occurrence of strokes in the IIT group. A third recent RCT found no mortality benefit and a much higher incidence of hypoglycemia in a group of patients treated to maintain glucose less than 108 mg/dL for 48 hours after cardiac arrest compared with a group treated to maintain glucose less than 144 mg/dL [26]. A prospective consecutive series of 818 patients admitted to a trauma ICU found no reduction in mortality or infectious complications in association with the implementation of a normoglycemic management protocol (glucose goal of 80 to 110 mg/dL) [47]. Additionally, preliminary results from another recently completed randomized trial of IIT in ICU patients showed no significant mortality difference and an increase in the risk of hypoglycemia [27,48].

The reason that IIT may result in increased mortality is unclear but may be related to a direct effect of insulin or to insulin-induced hypoglycemia. Two previous studies have found an association between ICU mortality and insulin administration [49,50]; the mechanism by which insulin may confer harm is not clear but might be related to anabolic effects, similar to growth hormone [51-53]. Two studies have examined the association between hypoglycemia and mortality in critically ill patients using case control methodology, with one finding no effect [54] and the other suggesting an independent association between severe hypoglycemia and mortality [55]. Further exploration of these areas is warranted.

It is unclear why we found increases in ICU mortality in the entire cohort and in some subgroups whereas there were only trends toward increased hospital mortality (Tables 4 and 5). For hospital mortality, the signal in the data could be attenuated due to the underlying noise of hospital deaths not related to glycemic control. It is also possible that ICU mortality reflects more glycemic control-related deaths than deaths from all causes. The association between period and ICU or hospital mortality was stronger in patients with a short ICU stay. This finding was also observed in the second randomized trial of van den Berghe and colleagues [24]. It is also possible, though speculative, that IIT confers some longer-term survival benefit that offsets adverse effects seen during the ICU stay.

## Conclusion

We observed that IIT in a mixed cohort of critically ill patients was not associated with a reduction in hospital mortality, and was associated with increased ICU and hospital mortality in some subgroups. These results, combined with data from the most recently concluded randomized trials, suggest that broad implementation of IIT may be premature and that additional randomized trials in diverse groups of critically ill patients are necessary.

## Key messages

• In a mixed population of critically ill patients, a large proportion of whom had suffered traumatic injury, intensive insulin therapy (IIT) was not associated with a reduction in adjusted hospital mortality.

• IIT was associated with an increase in intensive care unit (ICU) mortality and risk of organ failure after adjustment for baseline characteristics.

• The increase in adjusted ICU mortality was largest for the subgroup of patients admitted after trauma.

• Hospital and ICU mortality were increased in the subgroup of patients with an ICU length of stay of less than 3 days.

• The incidence of severe hypoglycemia increased approximately fourfold after implementation of IIT, although the incidence remained much lower than that reported in randomized trials of IIT.

## Abbreviations

APS = Acute Physiologic Score; CI = confidence interval; ICU = intensive care unit; IIT = intensive insulin therapy; LOS = length of stay; OR = odds ratio; RCT = randomized controlled trial; SAPS = Simplified Acute Physiology Score; SOFA = Sequential Organ Failure Assessment.

## Competing interests

The authors declare that they have no competing interests.

## Authors' contributions

MMT, VK, NDY, NW, and SAD were involved in the concept and design of the study, the analysis and interpretation of data, drafting the article or revising it critically for important intellectual content, and final approval of the version to be published. SD was involved in the concept and design of the study and in the analysis and interpretation of data. All authors read and approved the final manuscript.

## Appendix 1

Intensive insulin protocol used during period II of the study. These orders were modified slightly for use during period III in order to achieve a glucose goal closer to 81 to 110 mg/dL.
